# Bridge Model Updating Based on Wavelet Neural Network and Wind-Driven Optimization

**DOI:** 10.3390/s23229185

**Published:** 2023-11-14

**Authors:** Haifang He, Baojun Zeng, Yulong Zhou, Yuanyuan Song, Tianneng Zhang, Han Su, Jian Wang

**Affiliations:** 1 National Engineering Laboratory of Bridge Safety and Technology (Beijing), Research Institute of Highway Ministry of Transport, Beijing 100088, China; 2 Anhui Provincial Highway Management Service Center, Hefei 230022, China; lhy02027082@163.com; 3 School of Civil Engineering, Beijing Jiaotong University, Beijing 100044, China; 22125918@bjtu.edu.cn (Y.S.);; 4 School of Civil Engineering, Sun Yat-sen University, Guangzhou 510275, China

**Keywords:** finite element model updating, wavelet neural network, wind-driven optimization, surrogate model, bridge

## Abstract

Aging, corrosive environments, and inadequate maintenance may result in performance deterioration of civil infrastructures, and finite element model updating is a commonly employed structural health monitoring procedure in civil engineering to reflect the current situation and to ensure the safety and serviceability of structures. Using the finite element model updating process to obtain the relationship between the structural responses and updating parameters, this paper proposes a method of using the wavelet neural network (WNN) as the surrogate model combined with the wind-driven optimization (WDO) algorithm to update the structural finite element model. The method was applied to finite element model updating of a continuous beam structure of three equal spans to verify its feasibility, the results show that the WNN can reflect the nonlinear relationship between structural responses and the parameters and has an outstanding simulation performance; the WDO has an excellent ability for optimization and can effectively improve the efficiency of model updating. Finally, the method was applied to update a real bridge model, and the results show that the finite element model update based on WDO and WNN is applicable to the updating of a multi-parameter bridge model, which has practical significance in engineering and high efficiency in finite element model updating. The differences between the updated values and measured values are all within the range of 5%, while the maximum difference was reduced from −10.9% to −3.6%. The proposed finite element model updating method is applicable and practical for multi-parameter bridge model updating and has the advantages of high updating efficiency, reliability, and practical significance.

## 1. Introduction

Aging, corrosive environments, cracks caused by seismic activities, and inadequate maintenance may result in performance deterioration of civil infrastructures [1,2,3,4]; thus, employment of a structural health monitoring system to reflect the current situation is necessary to ensure both the safety and serviceability of structures. Finite element model updating is among the most common procedures in the structural health monitoring of civil engineering [5,6,7]. The traditional finite element model updating method utilizes the finite element model for calculation in each iteration when iteratively optimizing the updating parameters. The process is time-consuming, and the amount of calculation is huge; thus, it does not apply to engineering practice. Additionally, finite element models of complex structures require many parameters to be updated, the computational workload is large, and the traditional method is difficult to implement [8]. The method [9,10] based on surrogate models replaces the structural finite element model with a surrogate model with strong fitting ability, simple structure, and broad applicability and is combined with an optimization algorithm with strong global search ability and high efficiency to iteratively optimize the updating parameters. Thus, this method simplifies the model updating process and improves the efficiency of the updating work, and it is the most widely used method in the field of model updating [11,12,13,14].

The surrogate models commonly used in structural finite element model updating mainly include the polynomial response surface model [15,16,17], radial basis function (RBF) model [18,19], Kriging model [20], and neural network model [21,22,23,24,25]. Xu [26] proposed a bridge structure finite element model updating method based on the sparrow search algorithm (SSA) and the polynomial response surface method, and by updating the model of the high-dimensional locally damaged cantilever beam, the feasibility of the proposed method was verified. To more reasonably determine the number of node points of the Kriging surrogate model and to improve the prediction accuracy of the Kriging model for the extreme value region of the objective function, Qin [27] proposed an adaptive Kriging model and then applied it to the finite element model updating of bridge structures; the first-order frequency response surfaces of standard Kriging and adaptive Kriging fitting were established, and the finite element model updating of the two models was compared: The results show that the adaptive Kriging model can obtain better updating results. Qin [28] also combined the Kriging surrogate model with the improved gravity search algorithm (GSA), updated the initial finite element model using the load test data of the Nanzhonghuan Bridge, and obtained a reliable structural benchmark finite element model. Yang [29] employed the weighted response surface method to modify the single-girder finite element model of a bridge and adopted the central composite design experiment and variance analysis to screen out the parameters with high significance, thereby establishing a weighted response surface surrogate model; the objective function was constructed from the test load test data, and the structural parameters were identified by combining the chaotic particle swarm optimization algorithm; the obtained results are promising.

Among the models mentioned above, the neural network model has outstanding learning ability and widespread applicability [30], and it can directly update the structural parameters without solving the complex sensitivity matrix; in addition, it has a powerful nonlinear mapping function and strong robustness, which can fit the implicit function relationship between structural parameters and structural responses and better deal with data noise and ensure that the fitting results are not distorted [31,32]. Wavelet neural network (WNN) [33] is a product of the perfect combination of wavelet analysis theory and neural network theory. Based on the theory of neural networks, the wavelet function is used as the excitation function of the WNN. It inherits the superiority of wavelet analysis and artificial neural network, which has excellent approximation and fault tolerance, and is widely used in many engineering fields. However, the WNN lacks application in the finite element model updating of structures, and the related literature is scarce, but its excellent function approximation ability enables the network to be used as a surrogate model. Therefore, using the WNN as the surrogate model to update the finite element model has both research and application significance. This paper focuses on model updating and proposes a WNN combined with the WDO algorithm as the surrogate model of the finite element model.

In this paper, firstly, the theories of WNN and WDO and their realization are introduced; then, a structural finite element model updating method using a WNN as the surrogate model and WDO algorithm is proposed, and the method is implemented on the finite element model updating of Ningbo Bund Bridge; the updating parameters are studied and selected, and then, the updating procedure is carried out. It is demonstrated that the model updating method is applicable to the updating of the multi-parameter bridge model, which has practical significance in engineering and high efficiency in finite element model updating.

## 2. Wavelet Neural Network

In this section, the inception and concepts of wavelet neural network (WNN) are introduced, the structure of WNN is discussed, the training process of WNN is demonstrated conceptually, and finally, the shortcomings of WNN and measures to improve WNN are discussed.

Backpropagation (BP) neural network is the training process of feeding error rates back through a neural network to make it more accurate. The excitation function selected by the BP neural network is generally the Sigmoid function, which makes it difficult to guarantee the uniqueness of the solution, and the Sigmoid functions do not interact with each other; therefore, the learning convergence is slow. The RBF neural network needs to determine the center and width of the radial basis, and then, the linear optimization method is employed to determine the parameters, which are difficult to solve, and the radial basis function is non-orthogonal, which cannot guarantee the uniqueness of the approximation function. Wavelet neural network (WNN) was first proposed in 1992 [34] and was gradually developed into a mathematical modeling analysis method. WNN originated from the BP neural network [35]; however, it replaces the original excitation function Sigmoid function of the BP neural network with the wavelet function and obtains a new neural network similar to RBF neural network [36]. The excitation function of WNN is orthogonal or approximately orthogonal, which guarantees fast convergence and high fitting accuracy [37].

In addition, WNNs have the following characteristics [38]:

(1) The WNN can select different excitation functions for different fitting functions to improve the fitting;

(2) WNN can describe the function mutation stepwise, resulting in the function’s better fitting performance;

(3) Determining wavelet function and the whole network structure has a reliable theoretical foundation, which avoids blindness in network design.

### 2.1. WNN Structure

**Figure 1:**
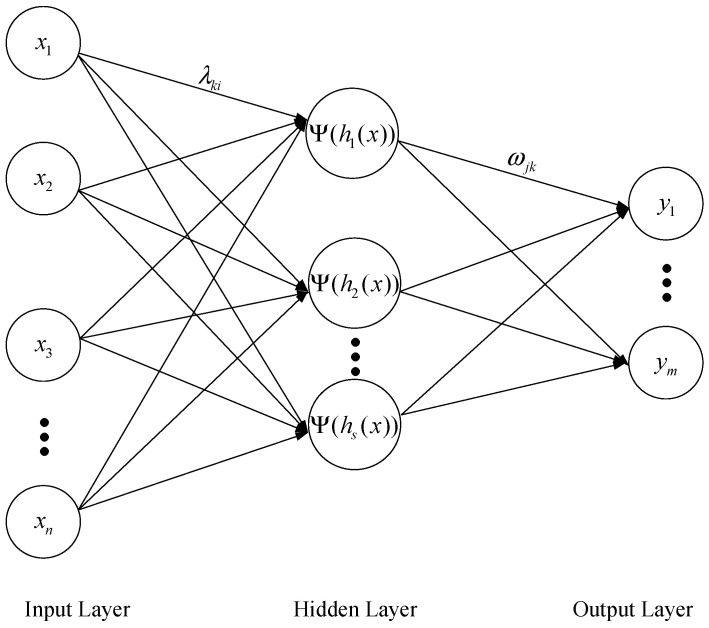
Network structure of WNN.

The WNN is consistent with the BP neural network structure, which consists of three layers: input layer, hidden layer, and output layer [39], as shown in Figure 1. The function of the input layer is to input sample data, the hidden layer uses the wavelet function $\Psi \left(t\right)$ as the excitation function, and the output layer can use the Sigmoid function and the linear Purline function as the excitation function.

Let {*x*_1_, *x*_2_, …, *x*_i_, …, *x*_n_} be the network input, {*y*_1_, *y*_2_, …, *y*_i_, …, *y*_n_} the network output, *n* the number of samples, and *m* the number of outputs; thus, the hidden layer can be defined as in Equations (1)–(4):

(1) $$\Psi \left(h_{k}\left(x\right)\right)=\Psi \left(\frac{\sum_{i=1}^{n}\lambda_{ki}x_{i}-b_{k}}{a_{k}}\right)\;=\Psi \left(\sum_{i=1}^{n}g_{ki}x_{i}+r_{k}\right)$$

(2) $$h_{k}\left(x\right)=\frac{\sum_{i=1}^{n}\lambda_{ki}x_{i}-b_{k}}{a_{k}}\;=\sum_{i=1}^{n}g_{ki}x_{i}+r_{k}$$

(3) $$g_{ki}=\frac{\lambda_{ki}}{a_{k}}$$

(4) $$r_{k}=-\frac{b_{k}}{a_{k}}$$

where *k* = 1, 2, …, *s*, *s* is the number of hidden layer nodes, $\lambda_{ki}$ is the connection coefficient between the input layer and hidden layer, $a_{k}$ is the k^th^ scaling factor, and $b_{k}$ is the k^th^ translation factor.

The output layer can be defined as Equation (5):

(5) $$y_{j}=f\left(\sum_{k=1}^{S}\omega_{jk}\Psi \left(h_{k}\left(x\right)\right)+\delta_{j}\right)\;=f\left(\sum_{k=1}^{S}\omega_{jk}\Psi \left(\sum_{i=1}^{n}g_{ki}x_{i}+r_{k}\right)+\delta_{j}\right)$$

where *f*(*x*) is the output layer excitation function, $\omega_{jk}$ is the connection coefficient between the hidden layer and the output layer, and $\delta_{j}$ is the neural network threshold. Similarly, for multiple samples, the hidden layer and output layer of the WNN can be defined as Equations (6)–(8):

(6) $$\Psi \left(h_{kt}\left(x\right)\right)=\Psi \left(\sum_{i=1}^{n}g_{ki}x_{it}+r_{k}\right)$$

(7) $$h_{kt}\left(x\right)=\sum_{i=1}^{n}g_{ki}x_{it}+r_{k}$$

(8) $$y_{jt}=f\left(\sum_{k=1}^{S}\omega_{jk}\Psi \left(\sum_{i=1}^{n}g_{ki}x_{it}+r_{k}\right)+\delta_{j}\right)$$

where *t* = 1, 2, …, *T*, *T* is the number of samples, $x_{it}$ is the *i*^th^ element of the *t*^th^ input samples, and $y_{jt}$ is the *j*^th^ neural network output, which corresponds to the *t*^th^ input samples. 

The matrix method is used to simplify the expression of hidden layer and output layer:

(9) $$X\left(n\right)=\left[\begin{array}{ccc} x_{11} & \cdots & x_{1T}\\ \vdots & \ddots & \vdots \\ x_{n1} & \cdots & x_{nT} \end{array}\right]$$

(10) $$I\left(s\right)=\left[\begin{array}{ccc} g_{11} & \cdots & \begin{array}{cc} g_{1n} & r_{1} \end{array}\\ \vdots & \ddots & \begin{array}{cc} \vdots & \vdots \end{array}\\ g_{s1} & \cdots & \begin{array}{cc} g_{sn} & r_{s} \end{array} \end{array}\right]$$

(11) $$V\left(m\right)=\left[\begin{array}{ccc} \omega_{11} & \cdots & \omega_{1s}\\ \vdots & \ddots & \vdots \\ \omega_{m1} & \cdots & \omega_{ms} \end{array}\right]$$

(12) $$W\left(m\right)=\left[\begin{array}{ccc} \omega_{11} & \cdots & \begin{array}{cc} \omega_{1s} & \delta_{1} \end{array}\\ \vdots & \ddots & \begin{array}{cc} \vdots & \vdots \end{array}\\ \omega_{m1} & \cdots & \begin{array}{cc} \omega_{ms} & \delta_{m} \end{array} \end{array}\right]$$

where $X\left(n\right)$ is the input sample matrix; $I\left(s\right)$ is the coefficient matrix consisting of $g_{ki}$ and $r_{k}$; $W\left(m\right)$ is the coefficient matrix consisting of $\omega_{jk}$ and $\delta_{j}$; $V\left(m\right)$ is the 1~n columns of $W\left(m\right)$, which is the coefficient matrix consisting of $\omega_{jk}$. Thus, we obtain the following:

(13) $$H\left(s\right)=\left[\begin{array}{ccc} h_{11}\left(x\right) & \cdots & h_{1T}\left(x\right)\\ \vdots & \ddots & \vdots \\ h_{s1}\left(x\right) & \cdots & h_{sT}\left(x\right) \end{array}\right]=I\left(s\right)\left[\begin{array}{c} X(n)\\ E_{1\times T} \end{array}\right]$$

(14) $$R\left(s\right)=\Psi \left(H\left(s\right)\right)$$

(15) $$Y\left(m\right)=\left[\begin{array}{ccc} y_{11} & \cdots & y_{1T}\\ \vdots & \ddots & \vdots \\ y_{m1} & \cdots & y_{mT} \end{array}\right]=f\left(W\left(m\right)\left[\begin{array}{c} R\left(s\right)\\ E_{1\times T} \end{array}\right]\right)$$

where $H\left(s\right)$ is composed of $h_{kt}\left(x\right),\;\;E_{1\times T}$ is the identity matrix of 1 row and *T* columns, $R\left(s\right)$ is the output matrix of hidden layer, and $Y\left(m\right)$ is the output matrix of neural network output layer. 

### 2.2. WNN Training

As a mature learning algorithm, gradient descent is often used to train neural networks. According to the idea of gradient descent and matrixing and introducing a momentum factor to accelerate the learning process, the iterative equations of coefficient matrices $W\left(m\right)$ and $I\left(s\right)$ are given:

(16) $${W\left(m\right)}_{p+1}=W{\left(m\right)}_{p}-\eta \frac{\partial U_{p}}{\partial {W\left(m\right)}_{p}}+a\left(W{\left(m\right)}_{p}-W{\left(m\right)}_{p-1}\right)$$

(17) $${I\left(s\right)}_{p+1}={I\left(s\right)}_{p}-\eta \frac{\partial U_{p}}{\partial {I\left(s\right)}_{p}}+a\left({I\left(s\right)}_{p}-{I\left(s\right)}_{p-1}\right)$$

where *p* is the number of iterations of the gradient descent method; $W{\left(m\right)}_{p}$ and ${I\left(s\right)}_{p}$ are the matrices obtained by the *p*-th iteration of $W\left(m\right)$ and $I\left(s\right)$; $U_{p}$ is the matrix of total error function of the neural network after the *p*-th iteration, that is, the performance function of the neural network; *η* is the learning rate; *a* is the momentum factor. To speed up the network training process and avoid falling into local optimum, the learning rate *η* needs to be adjusted, that is, the adaptive learning rate method. Using the adaptive learning rate to adjust the network can not only effectively improve the convergence rate but also improve the stability of the network learning process. The adjustment mechanism is as in Equation (18):

(18) $$\eta =\left\{\begin{array}{c} 1.05\eta \;\;\;\;\;\;\;\;U_{p+1}<U_{p}\;\;\;\;\;\;\\ 0.95\eta \;\;\;\;U_{p+1}>1.04\;U_{p}\\ \eta \;\;\;\;\;\;\;\;\;\;\;otherwise \end{array}\right.$$

The learning rate is adjusted by comparing the network’s total error function size before and after the update. When the error function increases, it indicates that the updating direction is wrong, or the updating amount is too large, and the learning rate needs to be reduced; when the error function decreases, it indicates that the direction is correct, and the learning rate should be increased.

Let $D\left(m\right)=\left[\begin{array}{ccc} d_{11} & \cdots & d_{1T}\\ \vdots & \ddots & \vdots \\ d_{m1} & \cdots & d_{mT} \end{array}\right]$ be the actual output sample; the mean squared error (MSE) function can be used as the network performance function:

(19) $$U_{p}=MSE=\frac{1}{T}{\left\|\left.D\left(m\right)-{Y\left(m\right)}_{p}\right\|\right.}_{2}^{2}=\frac{1}{T}\sum_{t=1}^{T}\sum_{j=1}^{m}{\left(d_{jt}-y_{jt,p}\right)}^{2}$$

Thus, we obtain the following:

(20) $$\frac{\partial U_{p}}{\partial W{\left(m\right)}_{p}}=\left(D\left(m\right)-{Y\left(m\right)}_{p}\right)⊙f\prime \left({W\left(m\right)}_{p}\left[\begin{array}{c} {R\left(s\right)}_{p}\\ E_{1\times T} \end{array}\right]\right)$$

(21) $$\frac{\partial U_{p}}{\partial {I\left(s\right)}_{p}}=\Psi \prime \left({H\left(s\right)}_{p}\right)\;⊙\left[{V{\left(m\right)}_{p}}^{T}\left(\left({Y\left(m\right)}_{p}-D\left(m\right)\right)\;⊙f^{\prime }\left({W\left(m\right)}_{p+1}\left[\begin{array}{c} {R\left(s\right)}_{p}\\ E_{1\times T} \end{array}\right]\right)\right)\left[\begin{array}{c} X\left(n\right)\\ E_{1\times T} \end{array}\right]\right]$$

where $y_{jt,p}$ is the element of $Y\left(m\right)$ in the matrix ${Y\left(m\right)}_{p}$ after the *p*-th iteration; ${V{\left(m\right)}_{p}}^{T}$ is the inversion of $V\left(m\right)$ after the *p*-th iteration; ${R\left(s\right)}_{p}$ is the matrix obtained after the *p*-th iteration of $R\left(s\right)$.

### 2.3. Improved WNN

The WNN still has shortcomings. For example, with the increase of the dimension of the problem, the number of training samples required by the network also increases sharply, which makes the network structure huge, and the learning and training convergence speed decreases; the number of hidden layer nodes of the network is difficult to determine, lacking scientific theoretical guidance; however, whether the selection of the number of hidden layer nodes is appropriate is directly related to the fitting performance and convergence accuracy of the network. The selection of the initial coefficient matrices ***W***(*m*) and ***I***(*s*) has a significant impact on the establishment and performance of the network, as an inappropriate initial coefficient matrix may lead to slow convergence of network errors, low convergence accuracy, or even non-convergence, resulting in an ill-conditioned network.

As for the problem of the dramatic increase of required training samples, it can be dealt with by sampling. The uniform design method (UDM) is employed to ensure the quality of the sample [40], and the sample experimental design approach is implemented at the expense of the minimal sample size to reduce the required number of samples.

As for the problem that the number of hidden layer nodes is difficult to determine since there is no conventional selection method for the number of hidden layer nodes, the empirical formula with mature application and universal applicability is employed to determine the number of hidden layer nodes, as in Equation (22) [41]:

(22) $$s=\sqrt{m+n}+\gamma$$

where γ is a constant in the range of 1 to 10.

**Figure 2:**
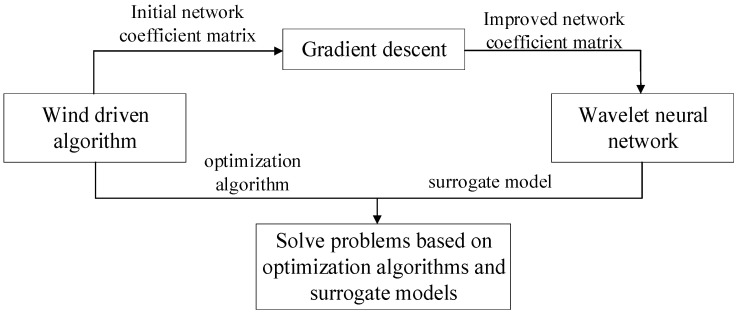
Combination of WDO and WNN.

To solve the problem of the selection of initial coefficient matrices ***W***(*m*) and ***I***(*s*), the wind-driven optimization (WDO) algorithm with strong robustness and high efficiency is used to select the initial coefficient matrix, thus improving the performance of the network. The combined use of the wind-driven algorithm and WNN is shown in Figure 2.

## 3. Wind-Driven Optimization Algorithm

In this section, the fundamentals of the algorithm of wind-driven optimization (WDO) are introduced, the update equations are derived, and finally, the realization of WDO is demonstrated.

WDO [42,43,44] is a novel-group iterative heuristic global optimization algorithm. The algorithm has the advantages of strong robustness and high optimization efficiency and is suitable for dealing with nonlinear, multi-extremal, and non-differentiable complex problems. It was first proposed in 2010 and has been rapidly developed and applied recently.

### 3.1. Fundamentals of the Algorithm

The motion of the atmosphere inspires WDO; that is, the atmosphere is considered to be composed of many air particles with a certain mass and volume, and the air particles satisfy the ideal gas law, so the motion state of each air particle can be described by Newton’s second law [45]. The fundamentals of the WDO algorithm are derivations of velocity and position update equations during the movement of air particles. Newton’s second law and ideal gas law are as Equations (23) and (24):

(23) $$\rho a=\sum_{i}F_{i}$$

(24) Q=ρRT

where ***a*** is the acceleration of air particles; *ρ* is the density of air particles in the atmosphere; $F_{i\;}$ is the different forces acting on the air particle; *Q* is the atmospheric pressure; *R* is the ideal gas constant; *T* is the atmospheric temperature.

The atmosphere moves on the earth’s surface and has a certain speed relative to the earth, usually called wind speed. When air particles move in a straight line with the wind speed vector in a rotating system, their inertia will produce a linear motion offset relative to the rotating system. Since Newton’s law is established in the inertial coordinate system, when dealing with the problem of the non-inertial rotating coordinate system, an imaginary force, the Coriolis force, needs to be introduced to describe the linear offset phenomenon in the rotating system. Combined with the physics of atmospheric motion, it is assumed that air particles are in a state of hydrostatic equilibrium, and the atmospheric motion is simulated by the four main forces acting on air particles, which are as follows:

(1) The pressure gradient force $F_{p}$ that makes the gas move from the high-pressure region to the low-pressure region is calculated by Equation (25):

(25) $$F_{p}=-\Delta Q\;\times \;\delta V=-\Delta \rho RT\times \;\delta V;$$

(2) The friction force $F_{u}$ that prevents the gas from flowing due to the pressure gradient force is calculated by Equation (26):

(26) $$F_{u}=-\rho \gamma u;$$

(3) The gravity force $F_{g}$ which is perpendicular to the center of the earth is calculated by Equation (27):

(27) $$F_{g}=\rho \times g\times \delta V;$$

(4) The Coriolis force $F_{c}$ describing the linear offset of air particles is calculated by Equation (28):

(28) $$F_{c}=-2\Omega \times u$$

where −ΔQ is the pressure gradient, and the negative sign indicates the direction of descent along the gradient; δV is the finite volume of air particles; γ is the friction coefficient; u is the wind velocity vector; ***g*** is the gravitational acceleration vector; Ω is the earth’s rotation angular velocity vector. The acceleration of air particles can be expressed as a=Δu/Δt, which can be obtained by substituting the four main forces into Newton’s second law as Equation (29):

(29) $$\rho \frac{\Delta u}{\Delta t}=F_{p}+F_{u}+F_{g}+F_{c}\;=\left(-\Delta Q\;\times \;\delta V\right)+\left(-\rho \gamma u\right)+\left(\rho \times g\times \delta V\right)+\left(-2\Omega \times u\right)$$

### 3.2. Derivation of Update Equations

To derive the velocity update equation, Equation (29) is simplified; let the air particle volume be δV=1, Δt=1. Both sides of the equation are divided by *ρ*, and then it is converted to ρ = ***Q***/*RT* according to the ideal gas law, which leads to the following:

(30) $$\Delta u=\frac{-\Delta Q\times RT}{Q}-\gamma u+g-\frac{2\Omega \times u\times RT}{Q}$$

In Equation (30), the gravity vector ***g*** in the three-dimensional coordinate system centered at the earth will change with the distance between the air particle and the center of the earth, and the WDO algorithm replaces the earth-centered three-dimensional coordinate system with a [−1, 1]-type one-dimensional coordinate system with 0 as the origin and assumes that the relationship between the vector ***g*** and the current position $x_{cur}$ of the air particle is ***g*** = $\left|g\right|\cdot (0-x_{cur})$. Therefore, the position of the air particle is also limited to the range of [−1, 1] so that the optimization range of the algorithm is [−1, 1].

**Figure 3:**
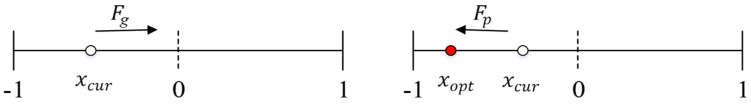
The friction force (*F_g_*) and pressure gradient force (*F_p_*) in the one-dimensional coordinate system.

Meanwhile, in the one-dimensional coordinate system, the direction of the pressure gradient force $F_{p}$ is from the high-pressure region to the low-pressure region, and the pressure value at the current position $x_{cur}$ of the air particle is defined as $Q_{cur}$, and the current minimum pressure value in the air particle swarm is $Q_{opt}$, which is the optimal pressure value, corresponding to the current optimal position $x_{opt}$ in the air particle swarm. Then, it can be assumed that the relationship between the pressure gradient −ΔQ and the position of air particle is $-\Delta Q\;=\left|Q_{opt}-Q_{cur}\right|\cdot \left(x_{opt}-x_{cur}\right)$. The gravity force $F_{g}$ and the pressure gradient force $F_{p}$ are shown in Figure 3.

In Equation (30), the velocity variable Δu can be expressed as $\Delta u=u_{new}-u_{cur}$, where $u_{cur}$ is the current velocity of air particle, and $u_{new}$ is the updated velocity of air particle. The friction force $F_{u}$ is affected by the current velocity; therefore, $u=u_{cur}$ is the friction force term, but the Coriolis force $F_{c}$ describes the influence of inertia on current velocity, and thus, the velocity $u_{cur}^{other\;dim}$ is used to describe the velocity of the current dimension that is affected by other dimensions; that is, $u=u_{cur}^{other\;dim}$ is the Coriolis force term, and this velocity ensures the balance between the algorithm’s exploration ability and development ability. Thereafter, Equation (30) can be further derived:

(31) $$u_{new}=\left(1-\gamma \right)u_{cur}-RT\left(x_{opt}-x_{cur}\right)\frac{\left|Q_{opt}-Q_{cur}\right|}{Q_{cur}}\;+gx_{cur}-\frac{2\Omega RTu_{cur}^{other\;dim}}{Q_{cur}}$$

To further improve Equation (31), let $=-2\left|\Omega \right|RT$. In addition, to make the WDO algorithm operational, the algorithm uses the ratio of air particle sorting numbers to replace the actual air pressure ratio; that is, $\frac{Q_{opt}}{Q_{cur}}=\frac{1}{k}$, where k is the sorting number obtained by sorting the air particles in ascending order according to the distance between the current position $x_{cur}$ and the current optimal position $x_{opt}$, and the sorting number for $x_{opt}$ is 1. Hence, the velocity update equation can be obtained as Equation (32):

(32) $$u_{\mathrm{new}}=\left(1-\gamma \right)u_{cur}-g\;x_{cur}+RT\left|\frac{1}{k}-1\right|\left(x_{opt}-x_{cur}\right)+\frac{{cu}_{cur}^{other\;dim}}{k}$$

where the recommended value ranges of correlation coefficients γ, *g*, *c*, and *RT* are shown in Table 1 [45].

Then, the position update equation of air particles is derived, and the relationship between position and velocity is as Equation (33):

(33) $$x_{new}=x_{cur}+u_{new}\times t$$

where $x_{new}$ is the update speed of air particles. Let *t =* 1; the equation can be simplified as in Equation (34):

(34) $$x_{new}=x_{cur}+u_{new}$$

Each updated air particle position is limited to the range of [−1, 1] due to the theoretical limit of the WDO algorithm. Meanwhile, its velocity must be limited to prevent the air particle position from moving too much. The limited update speed should be within the range of [${-u}_{max}{,u}_{max}$], where ${-u}_{max}\;$and $u_{max}$ are upper and lower bounds of air particle velocity.

The WDO algorithm takes the state of air particles as the research object and is an optimization algorithm established in a one-dimensional coordinate system, but the algorithm can map one-dimensional problems into N-dimensions to solve multi-dimensional optimization problems.

### 3.3. Realisation of WDO

A relational expression between the two needs to be established to relate the updating parameters of the structure to the position of the air particle. Let the updating range of the *i*-th updating parameter *p*_i_ be $\lambda_{1i}\%\sim \lambda_{2i}\%$; then, the range of *p*_i_ is $\left[p_{0i}(1+\lambda_{1i}\%),p_{0i}(1+\lambda_{1i}\%)\right]$, where *p*_0i_ is the initial value of *p*_i_. Suppose the position of an air particle (in N dimension) is ($x_{1}$, $x_{2}$, …, $x_{N}$), and the updating parameter of the structure is *p*_i_:

(35) $$\lambda_{1i}\%\leq \left(\frac{\lambda_{2i}+\lambda_{1i}}{2}+\frac{\lambda_{2i}-\lambda_{1i}}{2}\times x_{i}\right)\%\leq \lambda_{2i}\%$$

(36) $$p_{i}=p_{0i}\times \left(1+\left(\frac{\lambda_{2i}+\lambda_{1i}}{2}+\frac{\lambda_{2i}-\lambda_{1i}}{2}\times x_{i}\right)\%\right)$$

**Figure 4:**
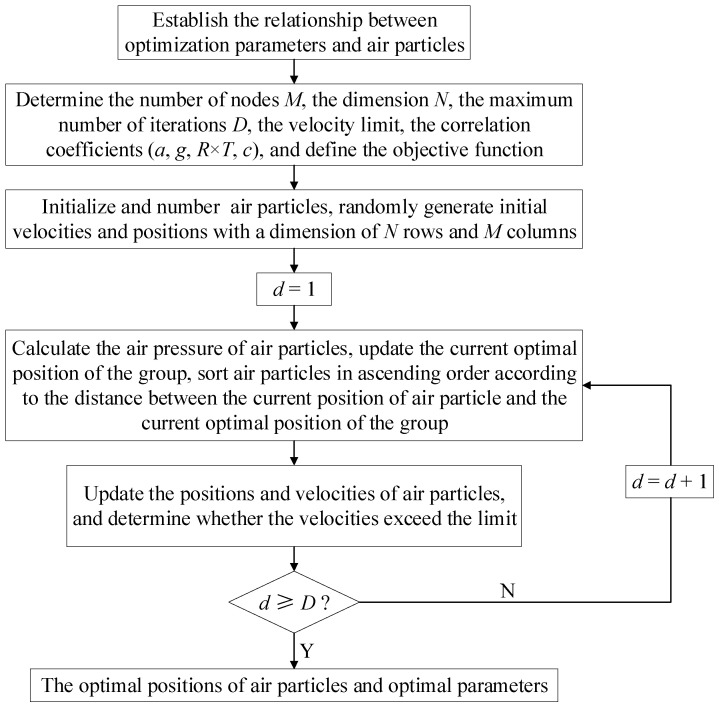
Calculation flow of WDO.

The WDO algorithm calculation flow is shown in Figure 4.

## 4. Verification of the Proposed Method

In this section, firstly, an initial finite element model that needs to be modified is established, and the model is regarded as the initial structure; secondly, the physical parameters in the model are adjusted appropriately, and the modified parameters are used as the physical parameters of the structure to establish a new finite element model, and the model is regarded as the actual structure; then, based on the difference in static and dynamic responses of the two models, the model is modified based on the WDO and CNN; finally, the updating results are compared and analyzed: If the difference between updated and actual values is slight, the model updating is completed. 

### 4.1. Finite Element Model

**Figure 5:**
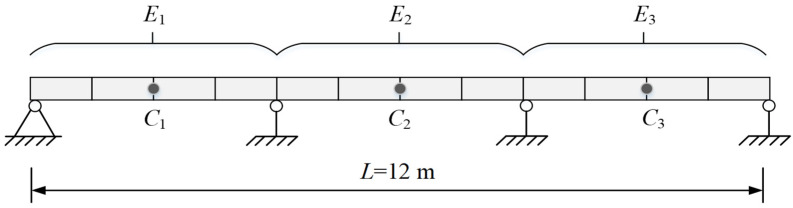
The structural representation of the finite element model.

The numerical model is a continuous beam structure of three equal spans, and the length of each span is 4 m. Since the elastic modulus and density of materials could change along with time, and their changes could influence structural behavior significantly, these data were therefore selected for verification and demonstration purposes. The elastic modulus of the material of the beam is *E* = 3.25 × 10^4^ MPa, and the density is *ρ* = 25 kN/m^3^. The beam’s cross-section is a rectangular section of 0.2 m × 0.15 m. The finite element model was established by finite element analysis software Midas Civil 2020, and the model was divided into 12 beam elements. The elastic moduli of each span of the three-span continuous beam are denoted as *E*_1_, *E*_2_, and *E*_3_ and the midpoint of each span as *C*_1_, *C*_2_, and *C*_3_. The finite element model is shown in Figure 5.

In practice, the material density of the structure may deviate, and the stiffness may change during service time. Therefore, for illustrative purposes, the material density *ρ* is assumed to be increased by 10%, and the elastic moduli *E*_1_, *E*_2_, and *E*_3_ are assumed to be decreased by 10%, 15%, and 20%, respectively, as the parameters of the actual structure. Values of these deviated parameters are called the actual values of the structure for simplicity, and the static and dynamic responses calculated by these deviated values are called the actual values of responses. The initial and actual values of parameters are shown in Table 2, where the difference is: (initial value − actual value)/actual value. 

The finite element model updating is carried out using the static and dynamic response of the structure, in which the dynamic response adopts the first six natural frequencies of the structure, and the static response is obtained by applying a vertical downward force of 30 kN at the two points C1 and C3 to obtain displacements at C1, C2, and C3. The initially calculated values obtained from the responses of the initial finite element model and the actual values of the finite element model with updated parameters are shown in Table 3. The difference in the table is: (initial value − actual value)/ actual value.

It can be seen from Table 3 that the initial values of the initial finite element model show considerable differences from actual values, and the differences in natural frequencies and displacements are both larger than 11%; therefore, the initial finite element model needs to be updated. According to the basic process of model updating, the four parameters of the initial model, *E*_1_, *E*_2_, *E*_3_, and *ρ* were updated, and for illustrative purposes, the objective function was constructed without conducting sensitivity analysis to screen the updating parameters.

Combined with static and dynamic responses of the finite element model, the objective function was constructed. The objective function takes the form of the residual sum of squares and is defined as Equation (37):

(37) $$U=\sum_{i=1}^{3}{\left(\frac{c_{i}^{t}-c_{i}^{a}}{c_{i}^{t}}\right)}^{2}+\sum_{j=1}^{6}{\left(\frac{f_{j}^{t}-f_{j}^{a}}{f_{j}^{t}}\right)}^{2}$$

where $c_{i}^{t}$ is the actual displacement at the midpoint *C_i_* of the *i*-th span, *i* = 1, 2, 3; $c_{i}^{a}$ is the initial displacement value; $f_{j}^{t\;}$ is the actual value of the *j*th order natural frequency, *j* = 1, 2, …, 6; $f_{j}^{a\;}$ is the initial value of the natural frequency. 

The four parameters to be updated were divided into 21 levels in the range of −10% to 10% with 1% as the step size, and the uniform design table [46] U_21_(21^4^) was used for experimental design. The parameter values of the 21 levels were sequentially brought into the initial finite element model, and the corresponding model responses were obtained. Taking 21 sets of parameter test values as input samples and taking the corresponding model responses as output samples, the input and output samples were normalized and used for WNN training.

### 4.2. Establishment of WNN

WNN parameters were selected tentatively by experience for verification purposes. The number of nodes in the network’s input layer is four, and the number of nodes in the output layer is nine. According to the empirical formula, the number of nodes in the network’s hidden layer was selected as 11. Meanwhile, the learning rate η of the network was selected as 0.01, and the momentum factor a is 0.5. In addition, the excitation function of the hidden layer adopts the Morlet wavelet function, and the excitation function of the output layer adopts the tansig function.

**Figure 6:**
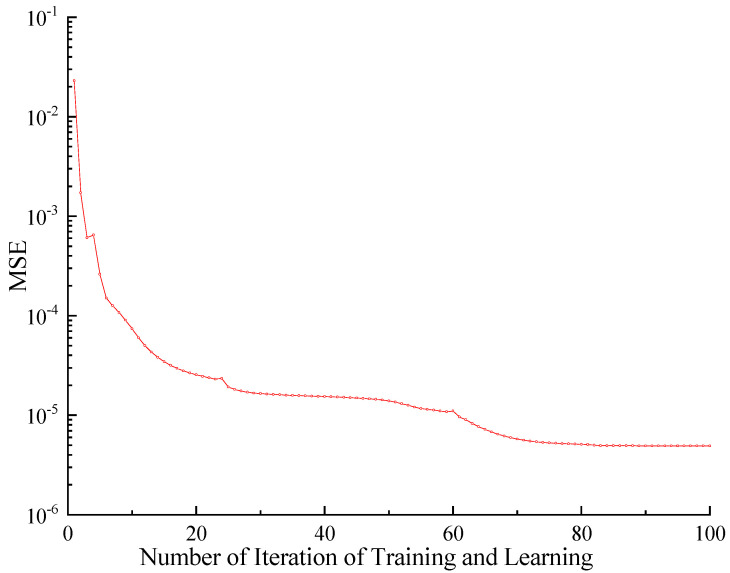
The training process of WNN.

When establishing the network, the WDO algorithm and the normalized test sample data are used to optimize the connection coefficient matrix of the WNN initially. Secondly, the WNN learning and training are carried out, and the MSE function is used as the network performance function. The learning and training process of the WNN is shown in Figure 6.

It can be seen from the figure that the WNN obtains a small network error in the process of 100 times of learning and training, and the MSE value is only 4.7 × 10^−6^. The small network error indicates that the WNN as a surrogate model of the finite element model has good fitting accuracy.

### 4.3. Results of Algorithm Optimization

**Figure 7:**
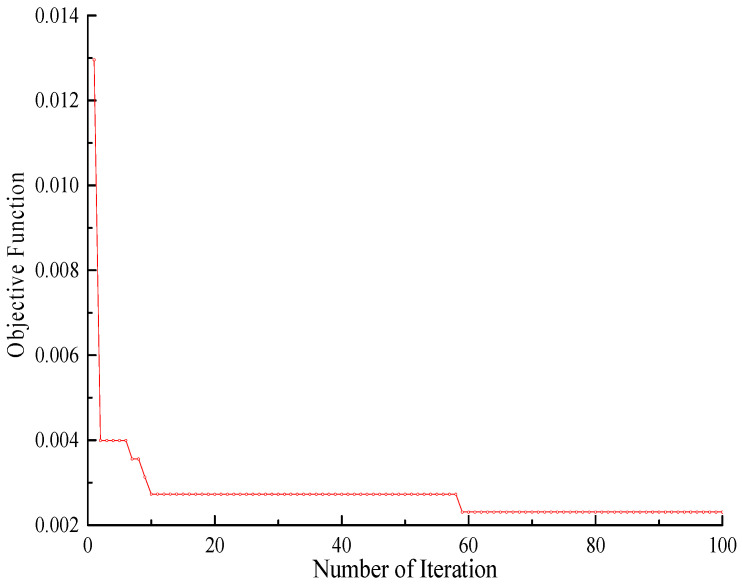
The iterative curve of WDO.

Combined with the WNN, the WDO algorithm optimizes parameters *E*_1_, *E*_2_, *E*_3_, and *ρ*. The four updating parameters correspond to the four dimensions of air particles, and 100 air particles are used for optimization calculation. The velocity limit is [−1, 1], the maximum number of iterations is 100, and the coefficients *a*, *g*, *RT*, and *c* in the velocity update equation are 0.85, 0.65, 0.5, and 2, respectively. The iterative curve of the objective function is shown in Figure 7.

It can be seen from the figure that the objective function converges at 58 iterations, and the objective function value is as small as 2.3 × 10^−3^, which shows that the optimization effect of the algorithm is obvious, and it is suitable for multi-parameter optimization. The corrected values of physical parameters are shown in Table 4.

The differences in updated and actual values are shown in Table 5. Based on the parameters, the WNN and the updated finite element model were used to calculate the structural response, respectively, to obtain the network value and the updated value of the structural response and then compared with actual responses. The comparison is shown in Table 6. Among them, the difference I is: (network value − actual value)/actual value; difference II is: (updated value − actual value)/actual value.

It can be seen from Table 5 that after updating parameters *E*_1_, *E*_2_, *E*_3_, and *ρ*, the differences become smaller, and the maximum difference is reduced from 25.00% to 3.92%. It can be seen from Table 6 that the values of difference I and difference II are both small, and the network value, updated value, and actual value of the static and dynamic responses are relatively close; thus, the model updating is proven to be effective. Therefore, the WNN can approximately reflect the nonlinear relationship between structural response and parameters and has good simulation performance; using the WNN as a surrogate model can effectively update the finite element model and improve work efficiency. 

## 5. Finite Element Model Updating of Ningbo Bund Bridge

In this section, the proposed is implemented on the model updating of Ningbo Bund Bridge, updating parameters are selected based on sensitivity analysis, WNN is established, and model updating is then carried out based on static and dynamic responses of the structure.

### 5.1. Introduction of Ningbo Bund Bridge

**Figure 8:**
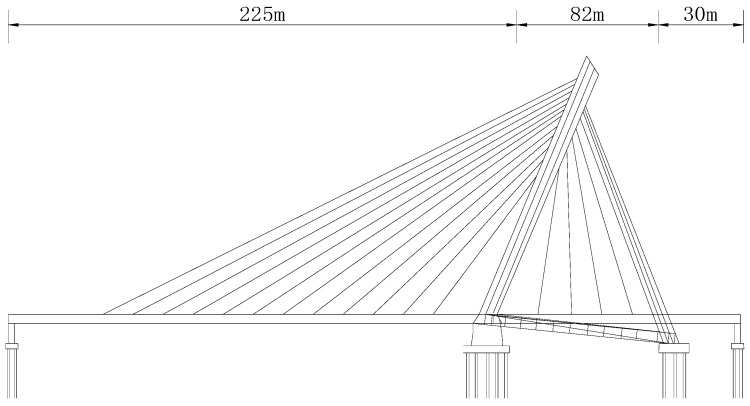
The elevation of Ningbo Bund Bridge.

The Ningbo Bund Bridge is a cable-stayed bridge made of steel with a single tower and four cable planes, as shown in Figure 8. 

The bridge’s total length is 337 m, and the span arrangement is (225 + 82 + 30) m from west to east. The main girder of the bridge adopts a separate steel box girder, which is connected by the cross-beam. The main girder adopts a varying cross-section along the longitudinal direction. The maximum beam height is 2.4 m, designed as a two-way six-lane bridge, and the standard width of the one-way is 21.4 m.

There are 64 stay cables in the bridge, including 46 main-span stay cables and 18 side-span stay cables. The stay cables are anchored on the head part of the front tower column and the inner and outer webs on both sides of the girders. The stay cables are anchored to the tower and inner and outer webs of the steel girder. The bridge tower adopts a triangular inclined tower structure composed of front tower columns, rear-inclined rods, horizontal rods, and other parts. The elevation of the bridge is shown in Figure 8.

### 5.2. Finite Element Model of Ningbo Bund Bridge

**Figure 9:**
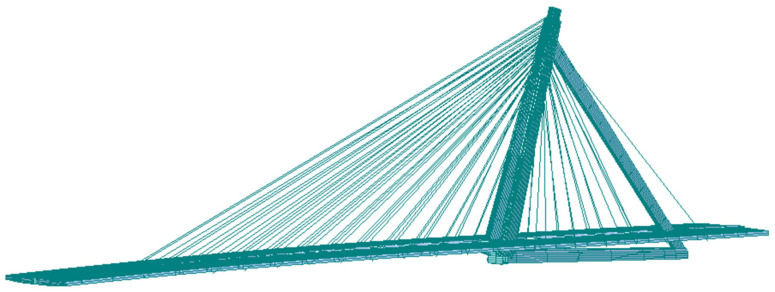
Finite element model of Ningbo Bund Bridge.

**Figure 10:**
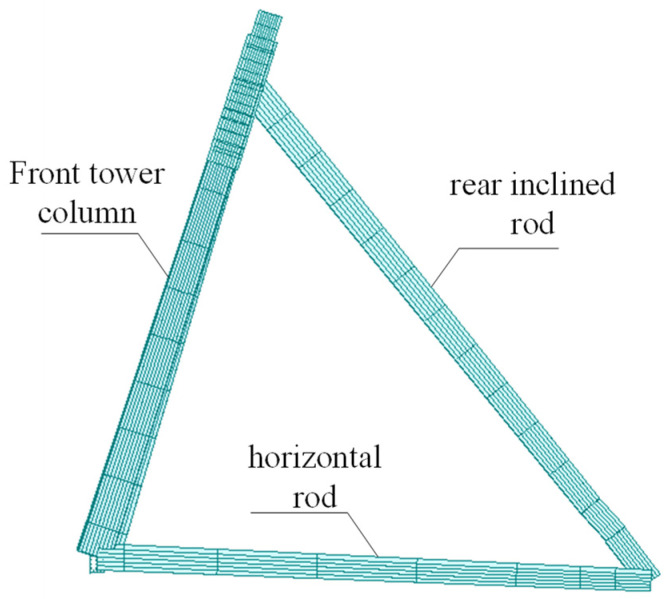
The schematic diagram of the triangular inclined tower structure.

According to design drawings and engineering construction practice, the finite element model of the bridge was established by the finite element analysis software Midas Civil 2020. The main girder and triangular inclined tower structure were simulated by the spatial beam element, the stay cable was simulated by the truss element, and the cable force was input by the measured cable force. The corresponding element mass adopts the equivalent density method, which is equivalent to the mass density of the element according to the mass of the segment, so that the structural mass of the finite element model is close to the actual mass of the structure. The tower and the bottom of the pier were consolidated to constrain all the degrees of freedom; the beam end restraint was simulated with spring elements to restrain the vertical and longitudinal displacements of the bridge. The established finite element model has a total of 309 nodes, 64 truss elements, and 193 beam elements, as shown in Figure 9. In the model, the triangular inclined tower structure is shown in Figure 10. The design load of the bridge is vehicle load highway-level I and city-level A, and pedestrian load is 2.4 kN/m^2^.

An ambient vibration test was carried out on the Ningbo Bund Bridge to obtain the structure’s measured frequencies and mode shapes. The ultra-low frequency (minimum sampling frequency up to 0.05 Hz) 991-type accelerometer was used in the test, the operation details in the experimental modal analysis are detailed in the literature [47], and a low-speed gear was selected for signal acquisition for low-frequency analysis. The measuring points were arranged in the main beam’s vertical, horizontal, and longitudinal direction, and the INV306D data signal acquisition and analysis system was used for data acquisition and modal analysis.

**Figure 11:**
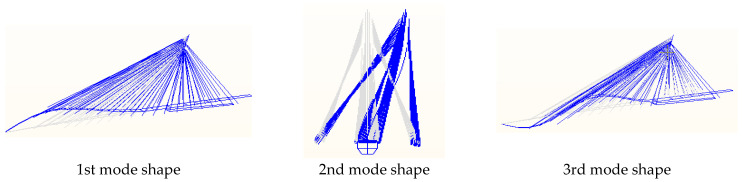
First three-order mode shapes of the bridge.

Meanwhile, the bridge’s initial finite element analysis was conducted to obtain the initial calculation results of the frequency and mode shape. The first three-order mode shapes are shown in Figure 11, and the first eight-order natural frequencies are shown in Table 7.

It can be seen from Table 6 that differences exist between the initially calculated value of the structural natural frequency and the measured value: The difference in first-order natural frequency is more than 10%, and the differences in the second-, fifth-, seventh-, and eighth-order natural frequencies are all above 5%; thus, the initial finite element model needs to be updated.

### 5.3. Selection of Updating Parameters

The selection of correction parameters is mainly based on engineering and modeling experience and sensitivity analysis methods. The Ningbo Bund Bridge has a novel structure and complex form and has many structural design parameters. When establishing the initial finite element model, the physical parameters of the structure, such as the elastic modulus of the material, mass density, geometric parameters, boundary conditions, and others, were mainly determined based on the construction design drawings, and these physical parameters are often different from the actual situation.

The Ningbo Bund Bridge is a steel bridge, and most components were prefabricated and assembled. Assembly may adversely affect the structural rigidity, and some detailed components may contribute to the structural rigidity to a certain extent. The influence of assembly and the contribution of detailed components to structural rigidity should be considered when establishing the model. Therefore, based on engineering experience, the elastic modulus of the main beam *E*_1_, the elastic modulus of the front tower column *E*_2_, the elastic modulus of the rear-inclined rod *E*_3_, the elastic modulus of the horizontal rod *E*_4_, the elastic modulus of the stay cable *E*_5_, and the elastic modulus of the cross-beam *E*_6_ were selected as updating parameters. Meanwhile, the equivalent density method was used in the modeling to equalize the mass of the detailed components, bridge deck pavement, and bridge deck auxiliary structures to the main structure, and there may be deviations in the mass conversion process. Therefore, the girder density *ρ*_1_, the front tower column density *ρ*_2_, the rear-inclined rod density *ρ*_3_, the horizontal rod density *ρ*_4_, the stay cable density *ρ*_5_, and the cross-beam density *ρ*_6_ were also selected as updating parameters. Additionally, the cable force of the bridge was adjusted according to the construction acceptance standard of the cable-stayed bridge, and a small cable force error was obtained; the error between the cable force of the completed bridge and the designed cable force is within 5% [48,49]. The whole bridge has a total of 64 cables, and to simplify the model updating process, as the cable force error is small, the actual measured cable force of the bridge was used as the input rather than selected as updating parameters. Furthermore, the error between the actual size and the design size of the prefabricated components of the bridge is very small, and the element section of the finite element model was established according to design drawings; thus, the geometric parameters of the sections were not considered. The boundary constraints of the bridge are few and all controlled in an ideal state, and the partially consolidated piers have little effect on the natural frequencies of the structure; hence, the boundary conditions were also not considered. 

**Figure 12:**
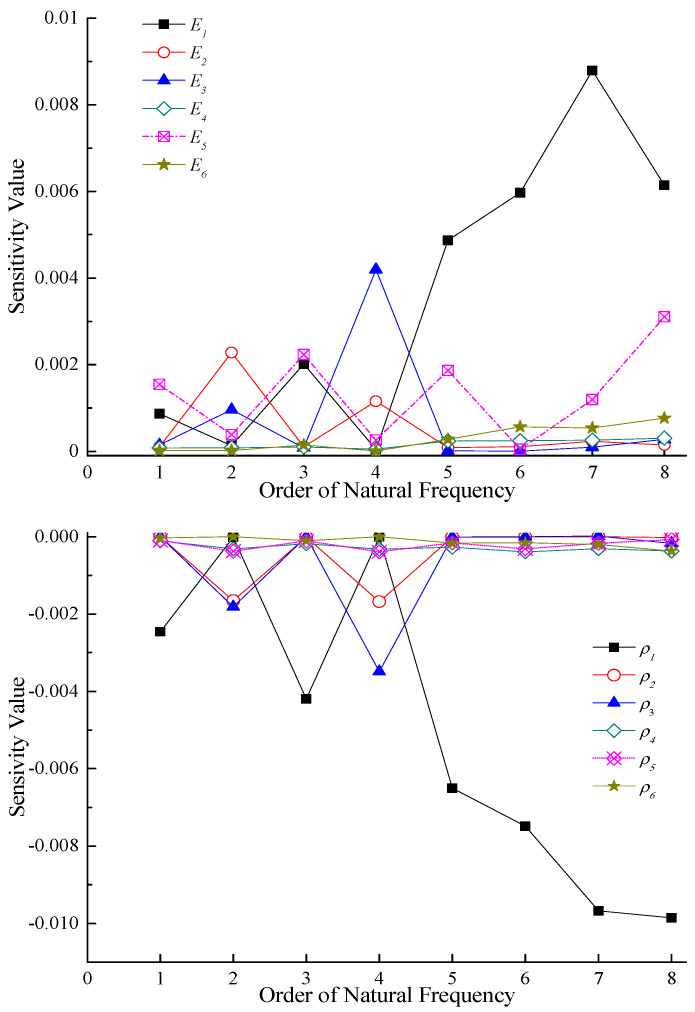
Sensitivity analysis of updating parameters.

When conducting parameter sensitivity analysis [50], each of the 12 updating parameters was individually increased by 1% and brought into the finite element model to approximately obtain the sensitivity values of each parameter corresponding to each order of natural frequency, as shown in Figure 12.

Through sensitivity analysis of 12 updating parameters, 7 parameters with large sensitivity values were finally selected as updating parameters, which include the elastic modulus of the main beam *E*_1_, the elastic modulus of the front tower column *E*_2_, the elastic modulus of the rear-inclined rod *E*_3_, the elastic modulus of the stay cable *E*_5_, the density of the main beam *ρ*_1_, the density of the front tower column *ρ*_2_, and the density of the rear-inclined rod *ρ*_3_. The initial values of the final updating parameters in the finite element model are shown in Table 8.

In the table, the elastic modulus of the main beam *E*_1_, the elastic modulus of the front tower column *E*_2_, the elastic modulus of the rear-inclined rod *E*_3_, and the elastic modulus of the stay cable *E*_5_ are all the elastic modulus values of steel: 2.06 × 10^4^ MPa; the density of the main beam *ρ*_1_, the density of the front tower column *ρ*_2_, and the density of the rear-inclined rod *ρ*_3_ are the values after mass conversion by the equivalent density method; hence, their densities are all greater than the density of steel, which is 7.85 × 10^3^ kg/m^3^.

### 5.4. Establishment of WNN

Seven updating parameters were divided into 31 levels within the range of −15% to 15% with a step size of 1%, and the uniform design table U31(31^7^) was employed for experimental design to obtain input samples. The parameters were brought into the initial finite element model of the bridge in turn, and the corresponding response output samples were obtained. Then, the input and output samples were, respectively, normalized and used as training samples of the neural network.

The number of nodes in the input layer of the network was set to seven and that of nodes in the output layer to eight; according to Equation (22), the number of nodes in the hidden layer of the network was selected as 15; the learning rate η of the network was selected as 0.01, and the momentum factor a was chosen as 0.5. In addition, the excitation function of the hidden layer adopts the Morlet wavelet function, and the excitation function of the output layer adopts the tansig function.

**Figure 13:**
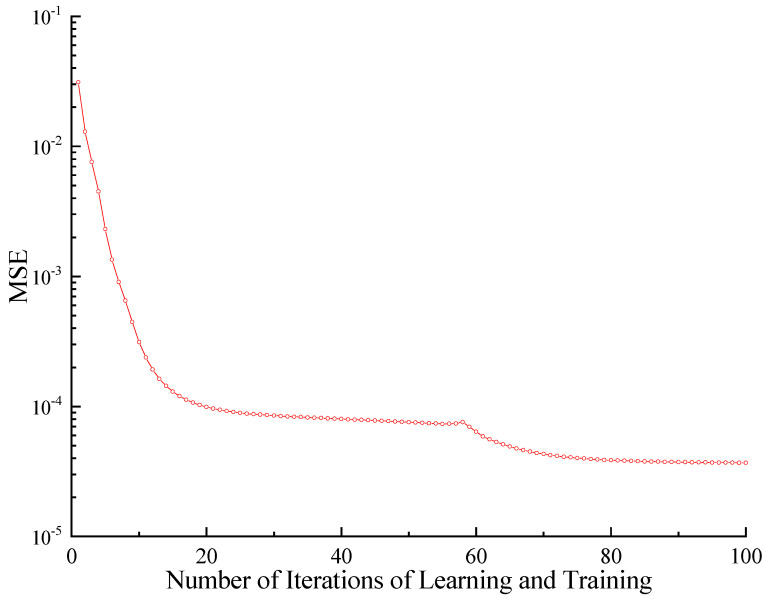
The training process of WNN.

When the network is established, the WDO algorithm and the normalized training samples are used to optimize the connection coefficient matrix of the WNN preliminarily. Secondly, the MSE function is adopted as the network performance function to train the WNN further. The learning and training process of the WNN is shown in Figure 13.

It can be seen from the figure that the WNN obtained a small network error after 100 times of training and learning, and the MSE value is only 3.7 × 10^−5^. The network training is efficient, and the network error is small; thus, it can be used as the surrogate model for the initial finite element of the bridge.

### 5.5. Results and Discussion

**Figure 14:**
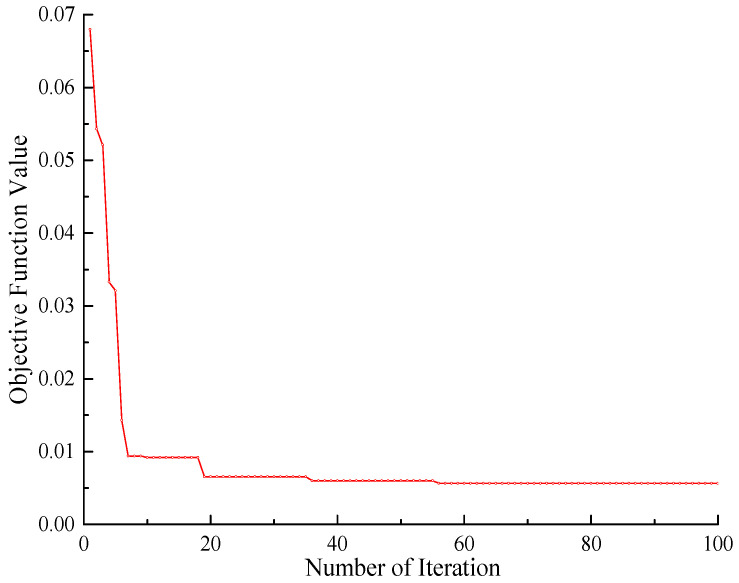
The iterative curve of WDO.

Combined with the well-trained WNN and WDO algorithm, seven parameters were updated. The seven updating parameters correspond to the seven dimensions of air particles. One hundred air particles were used for optimization calculation, and the velocity limit is [−1, 1], the maximum number of iterations is 100, and the coefficients *a*, *g*, *RT*, and *c* are, respectively, 0.85, 0.65, 0.1, and 2. The optimization process of the WDO algorithm is shown in Figure 14.

The objective function value reaches convergence in 57 iterations, and the convergence error is 5.6 × 10^−3^; thus, the optimization effect is obvious. The comparison between updated values and initial values is shown in Table 9. In the table, the difference is: (updated value − initial value)/initial value.

It can be seen from the table that the updating ranges of seven parameters are small, which are all within the range of −10~10%. Thus, it can be inferred that the decrease in values of *E*_1_ and *E*_2_ may be caused by the adverse effects of the structural assembly; the increase of *E*_3_ and *E*_4_ might be caused by the favorable effects of auxiliary structures and detailed components; the increase in the value of *ρ*_1_ is likely to be caused by the addition of bridge deck pavement load and auxiliary structures or the deviation caused by the mass conversion method during modeling; the decrease in the value of *ρ*_2_ may be caused by considering the extra mass of the detailed components in the initial finite element model or the deviation of the mass conversion method during modeling; the increase in the value of *ρ*_3_ may be caused by ignoring part of the mass of detailed components or the deviation of the quality conversion method during modeling. The selected seven updating parameters have reasonable changes in value, and the updated values of *ρ*_1_, *ρ*_2_, and *ρ*_3_ are all greater than 7.85 × 10^3^ kg/m^3^, which still lie in reasonable ranges.

The seven updated parameters were brought into the initial finite element model to obtain the updated value of the dynamic response of the structure. The comparison results with initial values are shown in Table 10. In the table, the difference is: (updated value − measured value)/measured value.

It can be seen from the table that after updating the selected seven parameters, the differences between the updated values and measured values all fell within the range of 5%, and the maximum difference was reduced from −10.9% to −3.6%; thus, the updating is proven to be effective. The finite element model updating method based on the WDO algorithm and WNN has proven to be applicable and practical for multi-parameter bridge model updating.

## 6. Conclusions

Finite element model updating makes a significant contribution to the healthy operation and damage detection of structures. This paper mainly focuses on model updating and proposes a WNN combined with the WDO algorithm as the surrogate model of the finite element model. The following conclusions are drawn:

(1) A structural finite element model updating method using a WNN as the surrogate model and WDO algorithm is proposed. The finite element model of the structure was updated based on static and dynamic responses of structures. The updated results show that the WNN can reflect the nonlinear relationship between the structural response and the parameters and has good simulation performance; the wind-driven optimization algorithm has the advantages of high optimization efficiency and fast convergence speed, and the method can effectively update the finite element model;

(2) The selection of updating parameters is important for improving the quality and efficiency of model updating. To avoid selecting unnecessary parameters, in this paper, the elastic modulus and mass density of the main beam and the elastic modulus and mass density of the triangular tower were preliminarily selected as updating parameters according to the engineering and modeling experience; then, sensitivity analysis was conducted, and the seven updating parameters were finally determined;

(3) The finite element model updating of the Ningbo Bund Bridge was studied. Combined with measured dynamic responses of the structure, the initial model was updated based on the WDO algorithm and WNN. The results show that the differences between measured dynamic responses and dynamic responses calculated by the updated finite element model were greatly reduced: The maximum difference was reduced from −10.9% to −3.6%. It can be concluded that the finite element model updating method based on the WDO algorithm and WNN is applicable and practical for multi-parameter bridge model updating and has the advantages of high updating efficiency, reliability, and practical significance.

For future research, as evidenced in Section 2 of the paper, since the fitting accuracy and stability of WNNs are affected by the initial value of coefficient matrix, the number of hidden layer nodes, the selected excitation function, the network learning method, and the number of hidden layers, currently, the information and scale of the network are often determined based on experience and trial calculation. How to correctly select the wavelet function, the number of hidden layers, and the number of hidden layer nodes still needs research.

## Figures and Tables

**Table 1:** Range of parameters [45].

Correlation Coefficient	γ	*g*	*c*	*RT*
Range	[0.8, 0.9]	[0.6, 0.7]	[0.05, 3.6]	[1.0, 2.0]

**Table 2:** Initial value and actual value of physical parameters.

Parameter	*E*_1_ (×10^4^ MPa)	*E*_2_ (×10^4^ MPa)	*E*_3_ (×10^4^ MPa)	ρ (×10^1^ kg/m^3^)
Initial value	3.250	3.250	3.250	2.500
Actual value	2.925	2.762	2.600	2.750
Difference/%	11.11	17.67	25.00	−9.09

**Table 3:** The initial value and actual value of the static and dynamic responses of the structure. (The positive sign indicates downwards displacement, and the negative sign indicates upwards displacement.)

Responses		Initial Value	Actual Value	Difference (%)
Natural frequency/Hz	1st order	15.151	13.295	13.96
	2nd order	19.383	17.040	13.75
	3rd order	28.203	24.788	13.78
	4th order	59.869	52.429	14.19
	5th order	67.678	59.568	13.61
	6th order	81.533	71.722	13.68
Displacement/mm	C1	−17.033	−19.243	−11.48
	C2	9.843	11.589	−15.07
	C3	−17.033	−20.926	−18.60

**Table 4:** The optimization results of WDO.

Parameter	Optimal Position of Air Particles	Corrected Value
*E*_1_/×10^4^ MPa	−0.444	2.889
*E*_2_/×10^4^ MPa	−0.576	2.782
*E*_3_/×10^4^ MPa	−0.674	2.702
*ρ*/×10^3^ kg/m^3^	0.470	2.849

**Table 5:** Differences in physical parameters.

Parameter	*E*_1_ (×10^4^ MPa)	*E*_2_ (×10^4^ MPa)	*E*_3_ (×10^4^ MPa)	ρ (×10^3^ kg/m^3^)
Updated value	2.890	2.782	2.702	2.849
Actual value	2.925	2.762	2.600	2.805
Difference/%	−1.22	0.74	3.92	1.57

**Table 6:** Comparison of structural responses.

Responses		Network Value	Updated Value	Actual Value	Difference I (%)	Difference II (%)
Natural frequency/Hz	1st order	13.415	13.274	13.295	0.90	−0.16
	2nd order	17.174	16.996	17.040	0.78	−0.26
	3rd order	24.973	24.714	24.788	0.75	−0.30
	4th order	52.996	52.416	52.429	1.08	−0.02
	5th order	59.955	59.377	59.568	0.65	−0.32
	6th order	72.214	71.453	71.722	0.69	−0.37
Displacement /mm	C1	−19.263	−19.361	−19.243	0.10	0.61
	C2	11.367	11.468	11.589	−1.91	−1.04
	C3	−20.082	−20.305	−20.926	−4.03	−2.97

**Table 7:** Measured and calculated values of natural frequencies.

Order of Natural Frequency	Measured Value (Hz)	Calculated Value (Hz)	Difference (%)	Description of Mode Shape
1	0.55	0.49	−10.9%	225 m span main girder vertical bending vibration
2	0.73	0.78	6.8%	Lateral vibration of the bridge tower
3	0.86	0.88	2.3%	225 m span main girder vertical bending vibration
4	1.26	1.21	−3.9%	Lateral bending vibration of the main beam
5	1.34	1.41	5.2%	Torsional vibration of the main beam
6	1.40	1.46	4.3%	Vertical bending vibration of the main beam
7	1.64	1.76	7.3%	Lateral vibration of the bridge tower
8	1.98	2.09	5.6%	Torsional vibration of the main beam

**Table 8:** Initial values of updating parameters.

*E*_1_	*E*_2_	*E*_3_	*E*_5_	*ρ*_1_	*ρ*_2_	*ρ*_3_
(×10^4^ MPa)	(×10^4^ MPa)	(×10^4^ MPa)	(×10^4^ MPa)	(×10^3^ kg/m^3^)	(×10^3^ kg/m^3^)	(×10^3^ kg/m^3^)
2.06	2.06	2.06	2.06	12.59	8.05	8.05

**Table 9:** Comparison between updated values and initial values.

Parameter	Updated Value	Initial Value	Difference (%)
*E*_1_/×10^4^ MPa	1.95	2.06	−5.3%
*E*_2_/×10^4^ MPa	1.92	2.06	−6.8%
*E*_3_/×10^4^ MPa	2.09	2.06	1.5%
*E*_5_/×10^4^ MPa	2.15	2.06	4.4%
*ρ*_1_*/*×10^3^ kg/m^3^	12.68	12.59	0.7%
*ρ*_2_*/*×10^3^ kg/m^3^	7.88	8.05	−1.9%
*ρ*_3_*/*×10^3^ kg/m^3^	8.43	8.05	4.7%

**Table 10:** Comparison of structural responses.

Structural Response		Updated Value	Measured Value	Difference (%)
Natural frequency/Hz	1st order	0.53	0.55	−3.6%
	2nd order	0.75	0.73	2.7%
	3rd order	0.87	0.86	1.1%
	4th order	1.22	1.26	3.2%
	5th order	1.35	1.34	0.7%
	6th order	1.44	1.40	2.9%
	7th order	1.69	1.64	3.0%
	8th order	2.01	1.98	1.5%

## Data Availability

Data is available upon request.
